# Supplementary material for: Coupling of ATPase activity, microtubule binding, and mechanics in the dynein motor domain
Source: EMBO J. 2019 May 31;38(13):e101414. doi: 10.15252/embj.2018101414 (PMC6600642; doi:10.15252/embj.2018101414)
Supplement: Supplementary file 1 — Appendix [file EMBJ-38-e101414-s001.pdf]

Appendix

for

Coupling of ATPase activity, microtubule binding, and mechanics in the dynein motor domain

Stefan Niekamp[1], Nicolas Coudray[2,3], Nan Zhang[1], Ronald D. Vale[1] & Gira Bhabha[2*]

[1] Department of Cellular and Molecular Pharmacology and Howard Hughes Medical Institute, University of California, San Francisco, 600 16th Street, San Francisco, CA 94158.

[2] Skirball Institute of Biomolecular Medicine and Department of Cell Biology, New York University School of Medicine, New York, NY 10016

[3] Applied Bioinformatics Laboratories, New York University School of Medicine, New York, NY 10016

* Corresponding author: gira.bhabha@gmail.com

This document includes:

- Appendix Figures (Appendix Fig. S1 - S8)
- Appendix Notes (S1-S3)
- Appendix Tables (Appendix Table S1 - S5)
- References for the Appendix

Appendix Figures

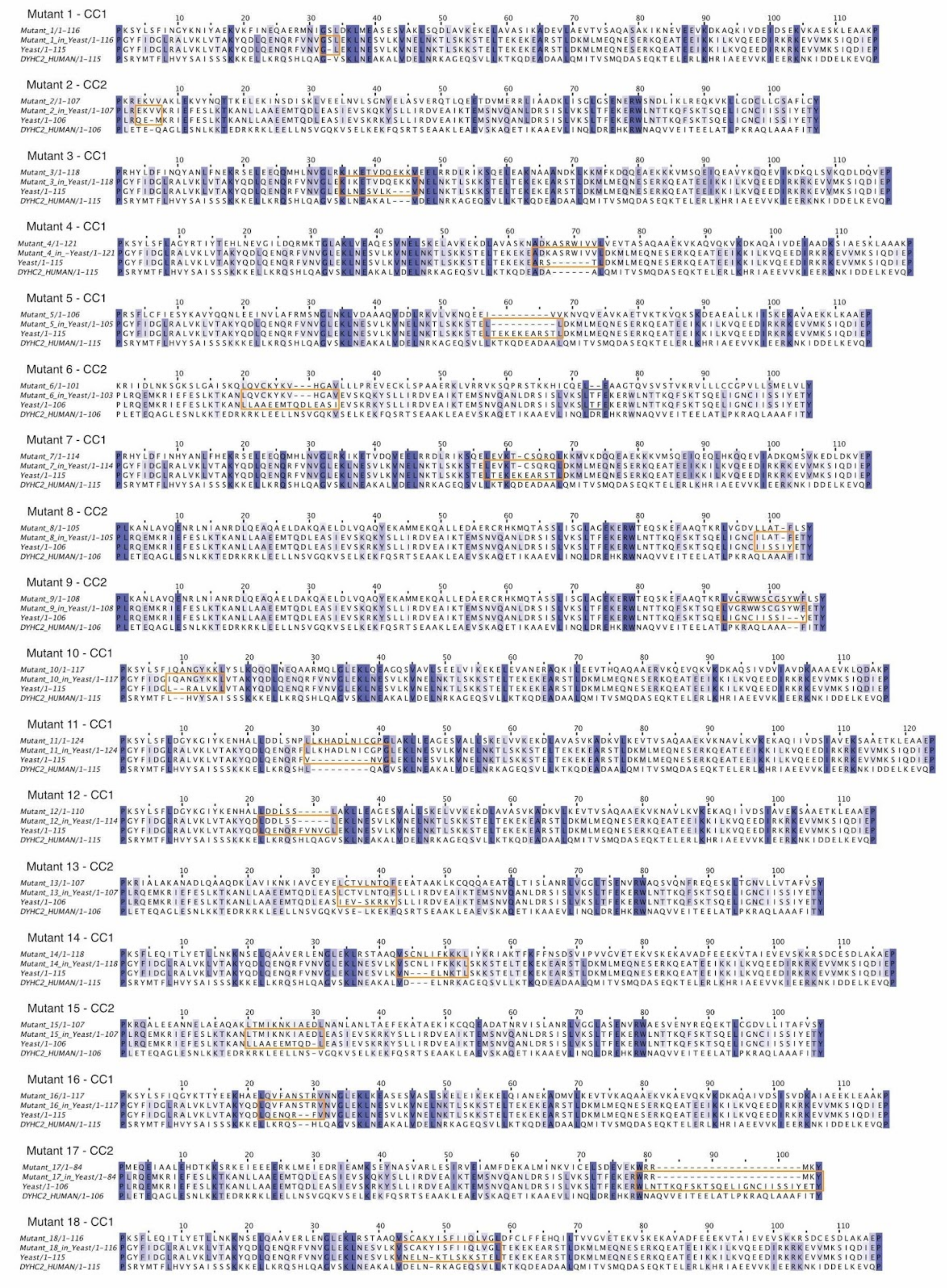

Appendix Figure S1. Sequence alignments of the panel of stalk mutants.

For each of the 18 mutants we compare the sequence of the species with insertion or deletion (top sequence), mutant created in yeast dynein background (second from the top), yeast dynein wild-type (second from bottom), and human cytoplasmic dynein 2 (bottom). Orange boxes highlight area of mutation. Note: Grey box in sequence alignment for mutant 6 shows second position of mutation for mutant 6 which was not created. Sequence conservation is indicated from white (not well conserved) to blue (highly conserved). For more details on how sequences were aligned and how mutants were selected see **Appendix Note S1**. The sequence alignment files used to create the mutants are available as **Appendix Source Data**.

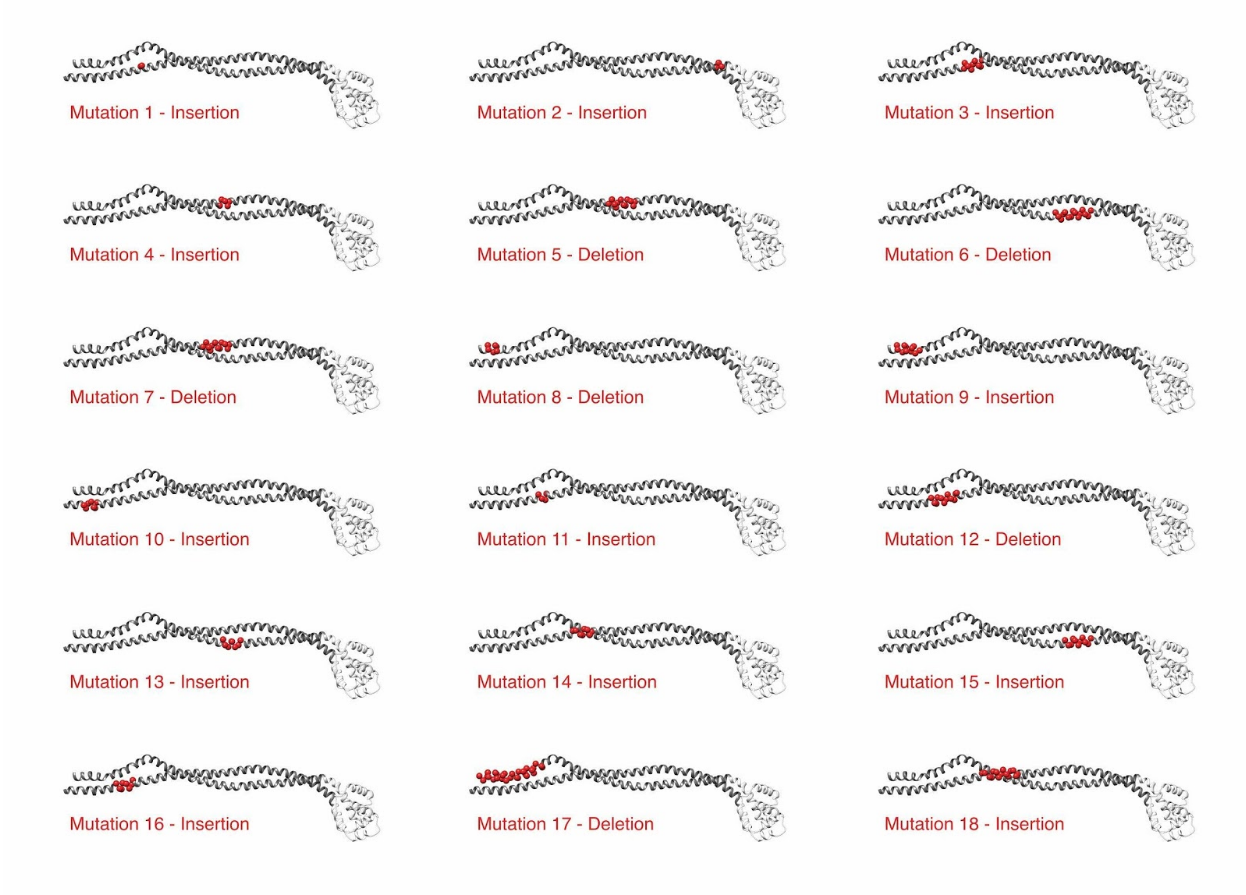

Appendix Figure S2. Position of all 18 insertion or deletion mutants mapped onto the structure of human cytoplasmic dynein 2 stalk (PDB: 4RH7 (Schmidt *et al*, 2015)**).**
Red spheres show residues that were altered in the stalk to either create an insertion or deletion.

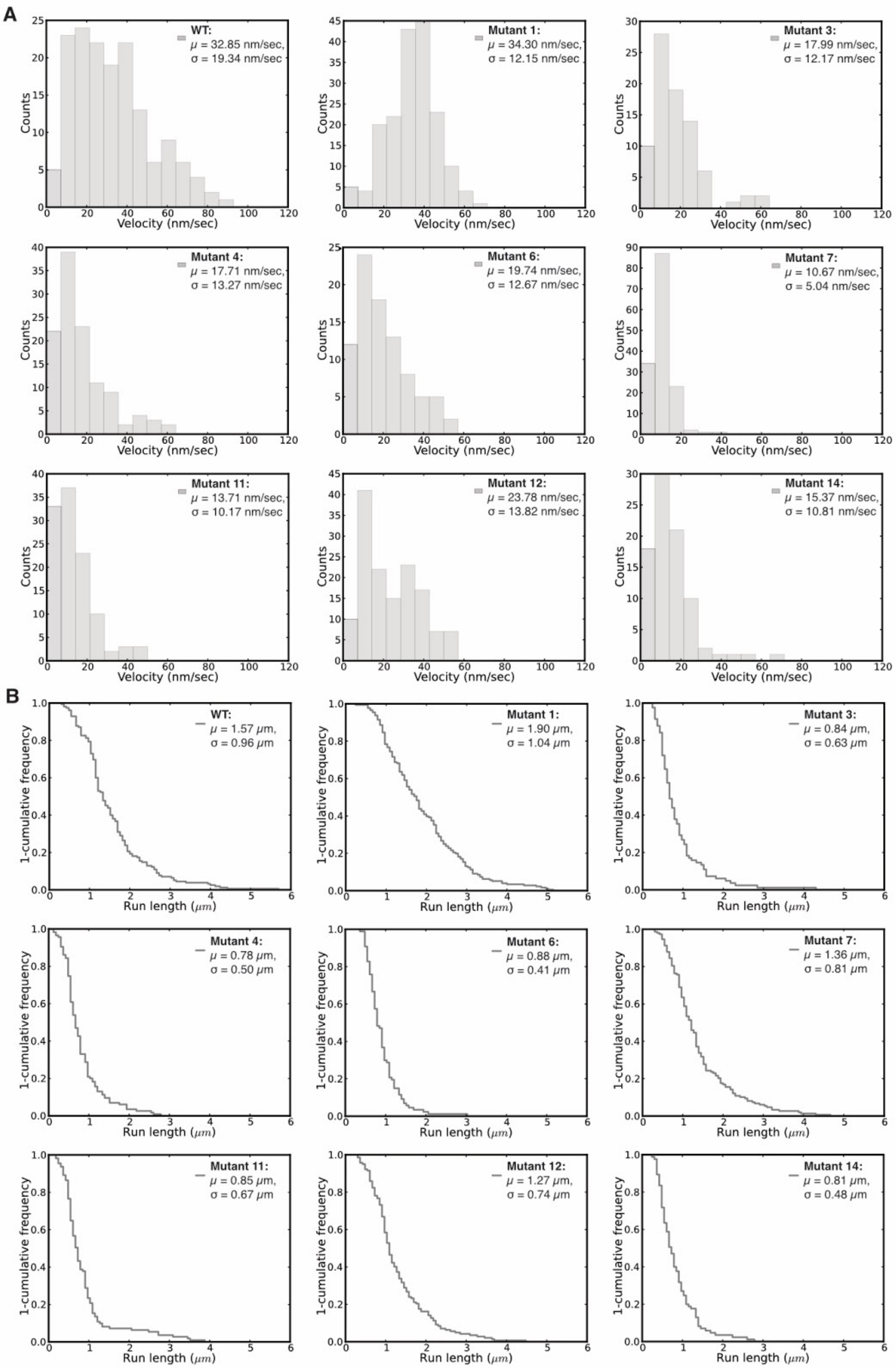
A
WT:
μ = 32.85 nm/sec,
σ = 19.34 nm/sec
Mutant 1:
μ = 34.30 nm/sec,
σ = 12.15 nm/sec
Mutant 3:
μ = 17.99 nm/sec,
σ = 12.17 nm/sec
Mutant 4:
μ = 17.71 nm/sec,
σ = 13.27 nm/sec
Mutant 6:
μ = 19.74 nm/sec,
σ = 12.67 nm/sec
Mutant 7:
μ = 10.67 nm/sec,
σ = 5.04 nm/sec
Mutant 11:
μ = 13.71 nm/sec,
σ = 10.17 nm/sec
Mutant 12:
μ = 23.78 nm/sec,
σ = 13.82 nm/sec
Mutant 14:
μ = 15.37 nm/sec,
σ = 10.81 nm/sec
Counts
Velocity (nm/sec)
B
WT:
μ = 1.57 μm,
σ = 0.96 μm
Mutant 1:
μ = 1.90 μm,
σ = 1.04 μm
Mutant 3:
μ = 0.84 μm,
σ = 0.63 μm
Mutant 4:
μ = 0.78 μm,
σ = 0.50 μm
Mutant 6:
μ = 0.88 μm,
σ = 0.41 μm
Mutant 7:
μ = 1.36 μm,
σ = 0.81 μm
Mutant 11:
μ = 0.85 μm,
σ = 0.67 μm
Mutant 12:
μ = 1.27 μm,
σ = 0.74 μm
Mutant 14:
μ = 0.81 μm,
σ = 0.48 μm
1-cumulative frequency
Run length (μm)

Appendix Figure S3. Single-molecule motility properties of wild-type like and transient binding stalk mutants.

A. Velocity histogram with average velocity (μ) and its standard deviation (σ) for wild-type, 'Transient binding', and 'Directional - WT like' mutants. Note: For mutant 14 only directional molecules were quantified and not the transient binding ones.

B. A '1-cumulative frequency distribution plot' of run length with average length (μ) and its standard deviation (σ) for wild-type, 'Transient binding', and 'Directional - WT like' mutants. Note: For mutant 14 only directional molecules were quantified and not the transient binding ones.

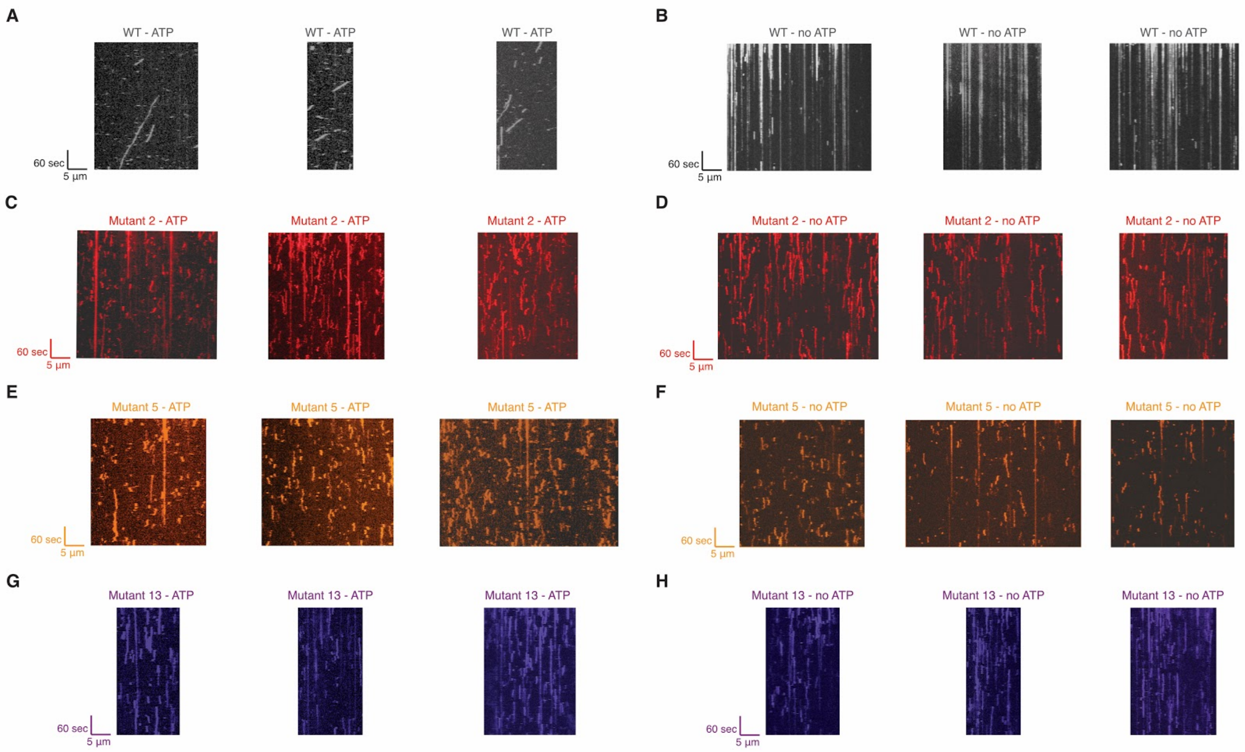

Appendix Figure S4. Kymographs of wild-type and diffusive mutants with and without ATP.

A, C, E, G Kymographs of wild-type (grey), mutant 2 (red), mutant 5 (orange), and mutant 13 (purple) with 1 mM ATP.

B, D, F, H Kymographs of wild-type (grey), mutant 2 (red), mutant 5 (orange), and mutant 13 (purple) without ATP.

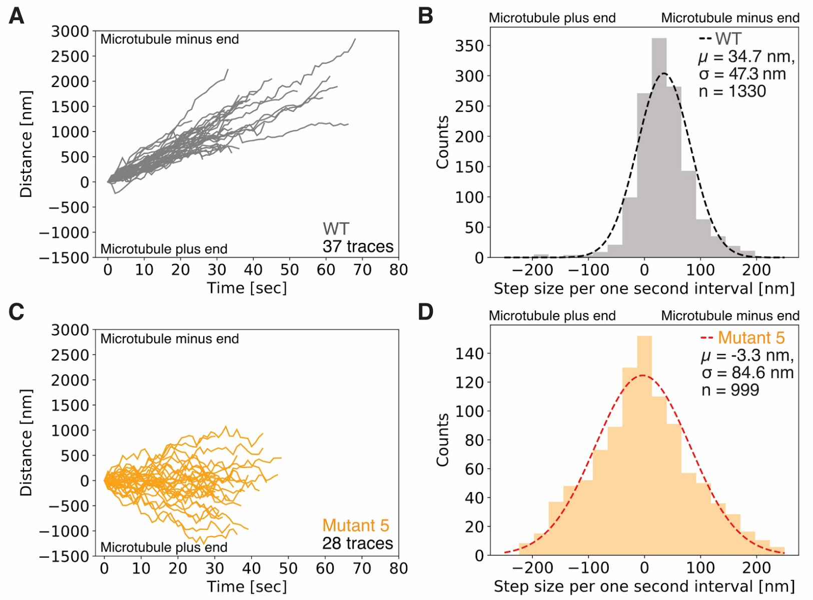

Appendix Figure S5. Quantification of movements of single molecules of wild-type and mutant 5 dynein.

A. Individual traces of wild-type dynein that were tracked using the 'localization microscopy' plug-in from µManager (Edelstein *et al*, 2010) (see **Materials and Methods**). Each trace is a single molecule that moved along a microtubule in the presence of 1 mM ATP. The traces in the panel were aligned so that all start at 0 nm and 0 sec. The polarity of microtubules was determined with human homodimeric kinesin-1 (K490) (Tomishige *et al*, 2006), which moves processively towards the plus end of microtubules.

B. Histogram of displacements of wild-type dynein per one second interval from all traces shown in **A**. Here, movement towards the microtubule minus end is a positive distance value while movement towards the microtubule plus end results in a negative distance value. Black dashed line is a Gaussian fit over the entire data.

C. Individual traces of mutant 5 that were tracked using the 'localization microscopy' plug-in from µManager (Edelstein *et al*, 2010) (see **Materials and Methods**). Each trace is a single molecule that moved along a microtubule in the presence of 1 mM ATP. The traces in the panel were aligned so that all start at 0 nm and 0 sec. The polarity of microtubules was determined with human homodimeric kinesin-1 (K490) (Tomishige *et al*, 2006), which moves processively towards the plus end of microtubules.

D. Histogram of displacements of mutant 5 per one second interval from all traces shown in **C**. Here, movement towards the microtubule minus end is a positive distance value while

movement towards the microtubule plus end results in a negative distance value. Red dashed line is a Gaussian fit over the entire data.

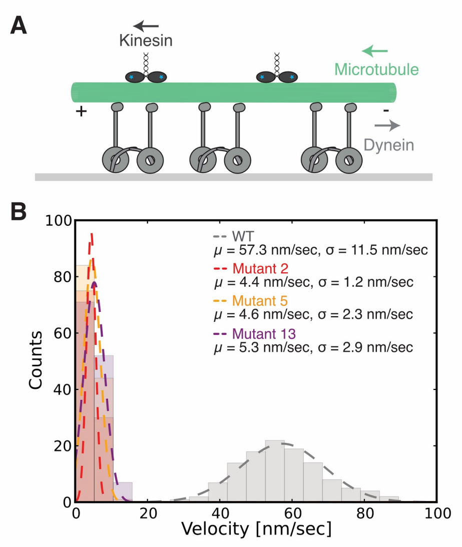

Appendix Figure S6. Gliding assay shows slow directional movement for mutants 2, 5, and 13.

A. Schematic of modified gliding assay. Dyneins (dimeric - dark grey) are immobilized on microscope slide (light grey) and can translocate microtubule (green). Plus end directed kinesins (dark blue) move on top of microtubule to mark directionality.

B. Histogram of gliding velocities of wild-type (grey, n=116), mutant 2 (red, n=105), mutant 5 (orange, n=129), and mutant 13 (purple, n=130) with average velocity (μ) and its standard deviation (σ). Example movies of microtubule gliding for all four constructs are shown in **Movies EV9-EV12**. Data of one dynein preparation is shown but a total of three repetitions of different dynein preparations resulted in very similar velocities.

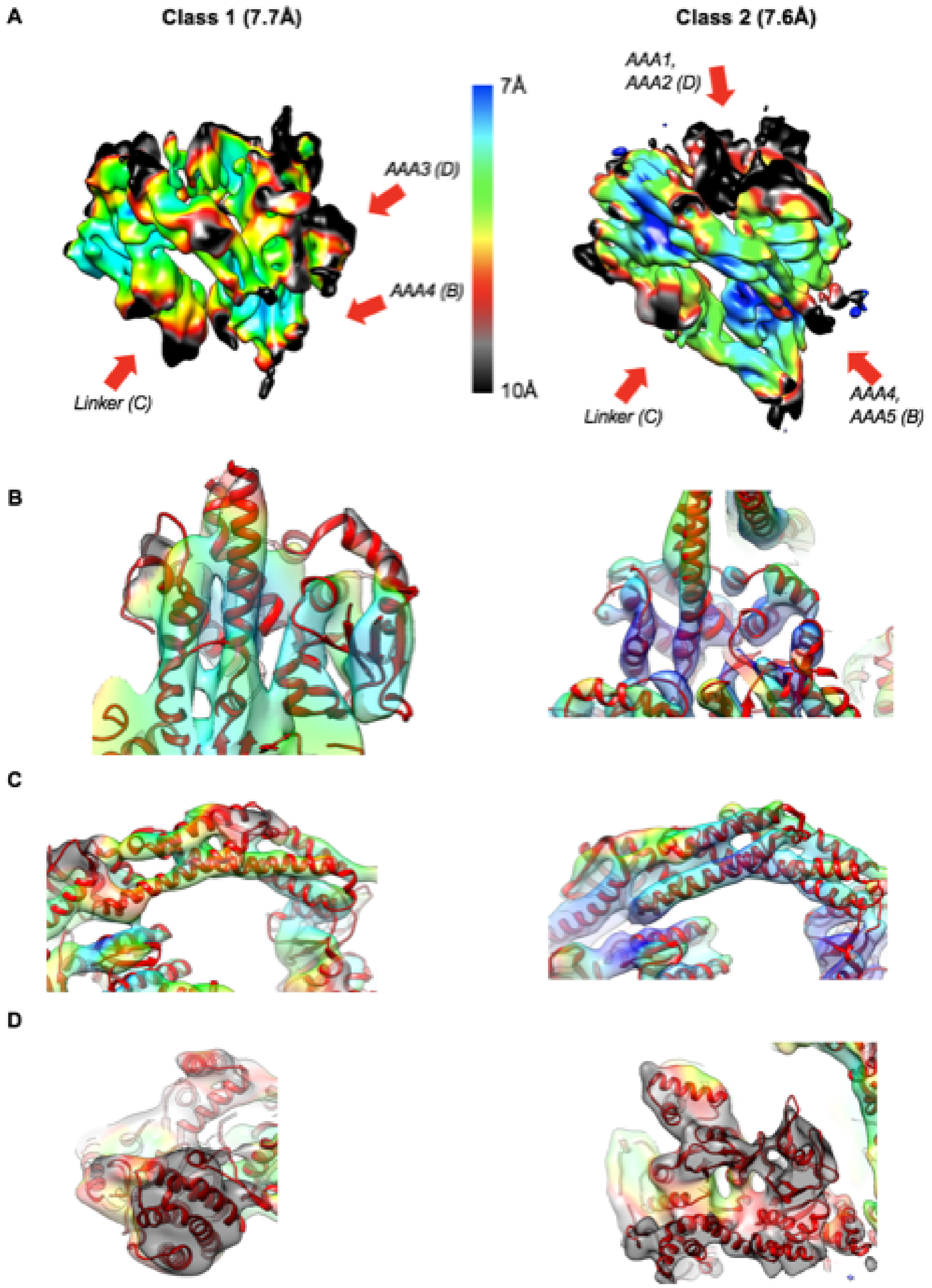

Appendix Figure S7. Local resolution of mutant 5 density map in AMPPNP.

A. The local resolution of mutant 5 in the presence of AMPPNP of class 1 (left) and 2 (right) is shown. The local resolution was estimated with CryoSparc v2.5.0. The red arrows indicate domains that are shown in a close-up view in **B-D**.

B-D Close-up view of different regions as indicated in **A** of well (**B**), medium (**C**) and poorly (**D**) defined parts of the density map with the PDB map docked-in.

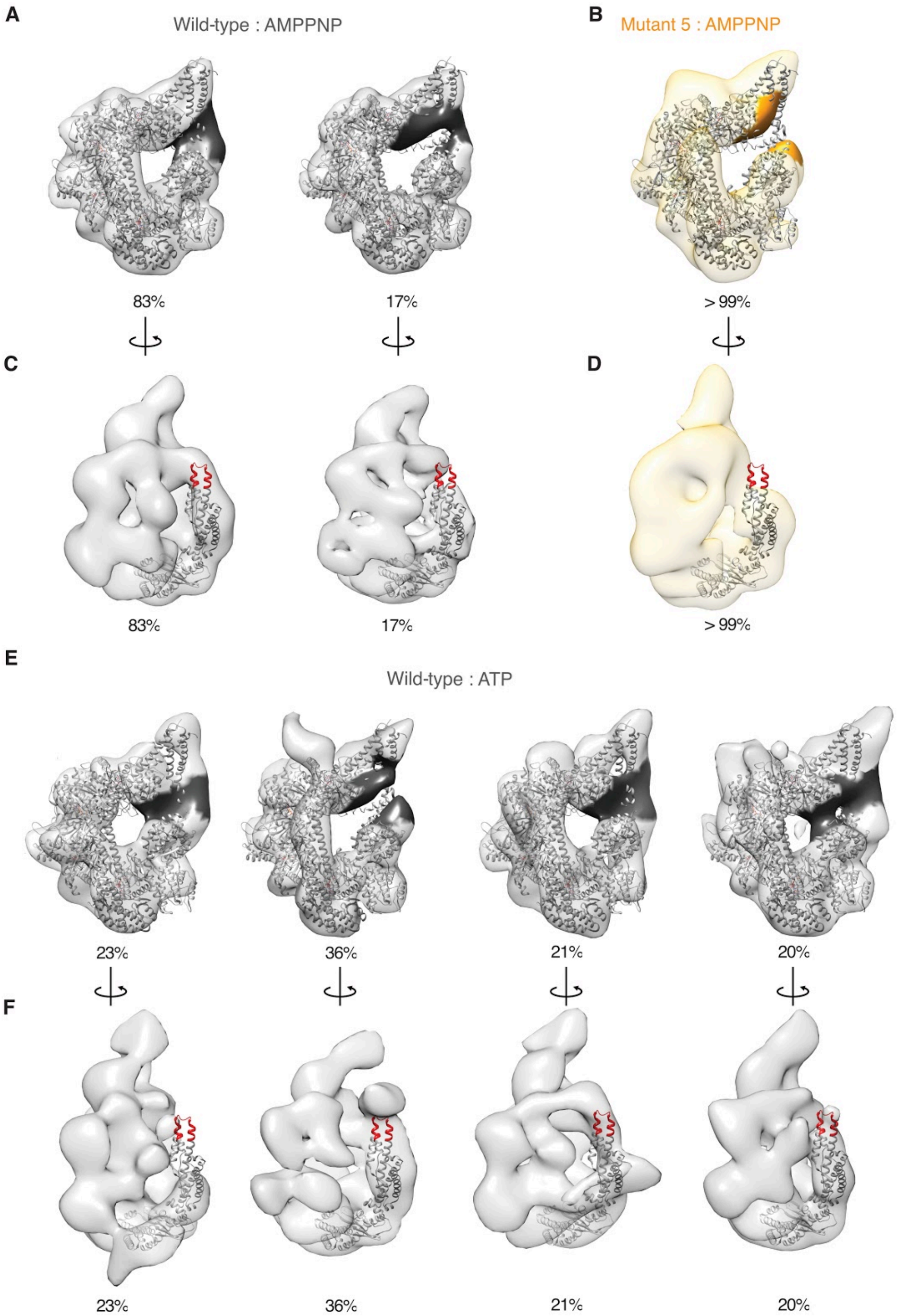

Appendix Figure S8. Negative stain reconstructions of mutant 5 and WT dynein.

A, C Negative stain EM reconstruction of wild-type dynein (grey) in the presence of AMPPNP (EMDB: 6064 and EMDB: 6063) with the AMPPNP crystal structure (PDB: 4W8F (Bhabha *et al*, 2014)) docked-in. Major (left, EMDB: 6064) and minor (right, EMDB: 6063) conformations are shown. These data was collected in a previous study (Bhabha *et al*, 2014).

B, D Negative stain EM density of mutant 5 (orange) in the presence of AMPPNP with the AMPPNP crystal structure (PDB: 4W8F (Bhabha *et al*, 2014)) docked-in.

A, B Area of weak density in the AAA5 region of minor wild-type conformation (dark grey) and for mutant 5 (bright orange) are highlighted.

C, D N-terminus of linker in crystal structure is highlighted in red and only linker and AAA1 of crystal structure are shown.

E, F Negative stain EM density data from a previous study (Bhabha *et al*, 2014) analyzed in the light of our new findings with the AMPPNP crystal structure docked-in (PDB: 4W8F) (Bhabha *et al*, 2014). **E.** Area of gap in density in the AAA5 region is highlighted in dark grey (EMDB: 6065-6068; from left to right, respectively). **F.** N-terminus of linker in crystal structure is highlighted in red and only linker and AAA1 of crystal structure are shown (EMDB: 6065-6068; from left to right, respectively).

Appendix Note S1

The coiled-coil stalk of the dynein motor domain is strikingly conserved in length

To better understand dynein's coiled-coil stalk, we obtained a dataset of 677 unique dynein heavy chain sequences from 229 fully sequenced eukaryotic genomes including sequences from cytoplasmic, axonemal and intraflagellar transport (IFT) dyneins. Since our analysis was focused on the motor domain and in particular on the stalk, we pruned our data set (see protocol for pruning below), and aligned 534 motor domain sequences using MAFFT (Katoh *et al*, 2002; Alva *et al*, 2016).

We defined boundaries of CC1 and CC2 of the stalk using anchor residues that are very well conserved: P2989 and P3101 define CC1, and P3228 and Y3333 define CC2 (**Fig. 1A**, residue numbers here and throughout the manuscript correspond to yeast cytoplasmic dynein, unless otherwise specified). When we first analysed the data set (sequences we used in this study were compiled in 2014), we noticed that the length of CC1 and CC2 are extremely conserved: 501 out of 534 sequences have exactly 115 residues in CC1 and 506 out of 534 sequences have exactly 106 residues in CC2 (**Fig. EV1H, I**). However, since the initial analysis was carried out, several sequences have been replaced with newer, corrected sequences which do not contain these insertions/deletions, suggesting that in reality closer to 1% of sequences deviate from the conserved stalk lengths (**Fig. EV1J, K, Appendix Table S1**). Furthermore, we note that the length and size of other subdomains of the dynein motor domain are not as well conserved (**Fig. EV1A, B**). Surprisingly, despite the conservation of length, the primary sequence of the stalk is not particularly well conserved (**Fig. EV1C, D**). We also investigated other features such as hydrophobicity and charge variation but no clear patterns of conservation were observed in the stalk (**Fig. EV1E-G**).

Our initial analysis revealed a handful of sequences that varied in length from 115 residues in CC1 and 106 residues in CC2 (**Fig. EV1H, I**). Coiled-coils contain a repeating pattern of 7 residues (heptad repeat) consisting of charged and hydrophobic residues. The hydrophobic stretch of the heptad repeat in one coil interacts with the hydrophobic region of the heptad repeat on the other coil, thus generating a stable coiled-coil. Based on the conserved structural motifs in the coiled-coil, one may expect that, for example, a deletion of 7 residues in CC1 would correspond to a deletion of 7 residues in CC2, in order to maintain the interaction between the two coils. Indeed, previous work has shown that insertions and

deletions of the same number of residues in both sides does not alternate velocity and ATPase activity significantly (Carter *et al*, 2008). Surprisingly, however, the variants in our dataset contained either insertions or deletions in one of the coils, but not in both simultaneously.

Interestingly, the sequences of all three mutants that showed diffusive movement have been updated in the databases and do not show any insertions or deletions anymore. Based on currently available sequences, most likely dyneins with this phenotype do not exist; however, we serendipitously stumbled upon these insertions and deletions in regions of the stalk, which do mediate communication between the AAA ring and MTBD, and these mutations shed light on the dynein motility mechanism.

Protocol for sequence alignments and design of stalk mutants

All files listed in the following format are available to download from the **Appendix Source Data**:

--- *Sequence-alignment.fasta* ---

Initial dataset:

677 unique dynein heavy chain sequences (axonemal and cytoplasmic) from 229 fully sequenced eukaryotic genomes (from Christian Zmasek, Godzik lab, Burnham)

--- *All-677-heavy-chain-sequences_MAFFA-alignment.fasta* ---

Pruning of initial dataset:

1. To focus on the motor domain, we eliminated tail sequences and used sequences starting at E1364 (yeast numbering)
2. We removed sequences with ambiguity in sequencing reads (i.e. contains “X” in sequence)
3. We removed incomplete sequences (e.g. no amino acids in AAA6 domain)
4. We used MAFFT on the MPI Bioinformatics Toolkit server (Alva *et al*, 2016; Katoh *et al*, 2002) to realign the remaining sequences with a ‘Gap open penalty’ of 1.53 and an ‘Offset’ of 0.0
5. We removed sequences that didn’t contain a functional Walker A motif in AAA1 (mutation in K1802 - yeast numbering)

6. We deleted sequences that didn't have a functional Walker B motif in AAA1 (mutation in E1849 - yeast numbering)
7. We again used MAFFT on the MPI Bioinformatics Toolkit server to realign the remaining sequences with a 'Gap open penalty' of 1.53 and an 'Offset' of 0.0

Final dataset:

534 unique dynein heavy chain sequences

--- *Pruned-heavy-chain-sequences_MAFFA-alignment.fasta* ---

Division into CC1 and CC2 stalk sequences:

1. Based on conserved residues in the multiple sequence alignment (MSA) file we used P2989 to P3103 for CC1 and P3228 to Y3333 for CC2 anchor points (yeast numbering)
2. We then truncated the final MSA file to CC1 only, to CC2 only and to CC1-MTBD-CC2 only
3. We realigned all three MSA using MAFFT on the MPI Bioinformatics Toolkit server with a 'Gap open penalty' of 1.53 and an 'Offset' of 0.0
   a. --- *Pruned-heavy-chain-sequences_CC1_2989-3103_MAFFA-alignment.fasta* ---
   b. --- *Pruned-heavy-chain-sequences_CC1-MTBD-CC2_2989-3333_MAFFA-alignment.fasta* ---
   c. --- *Pruned-heavy-chain-sequences_CC2_3228-3333_MAFFA-alignment.fasta* ---

Identification of outlier sequences:

As can be seen in **Fig. EV1** most dynein sequences of CC1 and CC2 have a well defined length of 115 and 106, respectively. Thus, we first removed all outlier sequences, these are ones that are not 115/106 amino acids long and created new alignment files for CC1 and CC2 using MAFFT on the MPI Bioinformatics Toolkit server with a 'Gap open penalty' of 1.53 and an 'Offset' of 0.0. This left us with 476 sequences. We then added one outlier sequence back at a time and created unique alignments for all possible mutations by MAFFT on the MPI Bioinformatics Toolkit server with a 'Gap open penalty' of 1.53 and an 'Offset' of 0.0.

--- *Folder: Mutants with alignments: i.e. Mutant-01_CC1.fasta* ---

Selections of insertions / deletions to clone:

We decided to clone mutations (insertion / deletion) that were between well defined anchor points (highly conserved residues - see Figure below (based on PDB: 4RH7) (Schmidt *et al*, 2015)), which allowed us to generate the mutant accurately in the yeast dynein background.

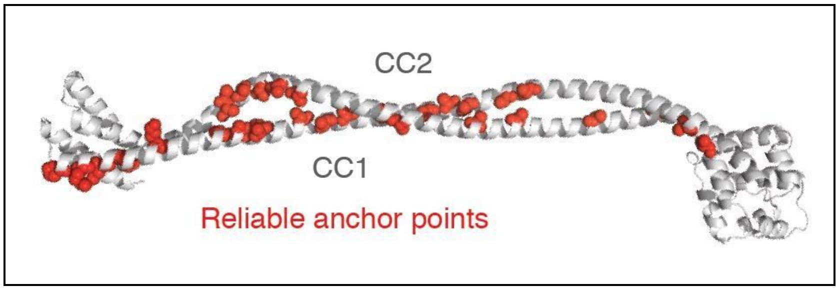

The identification of reliable anchor residues indicates regions of high sequence conservation in the stalk. In regions where reliable anchor residues were not identified, the sequence was more variable, and thus precluded us from designing meaningful insertion/deletion mutants in these regions with confidence, even though some outlier sequences did show insertions and deletions around these regions. Similar to the initial data set, our 18 mutants arise from various species and include cytoplasmic as well as axonemal dynein sequences (**Appendix Table S1**).

Updated information with newly deposited sequence data:

We checked the sequence databases again for the sequences that have insertions or deletions and found that many were updated and corrected in the meantime (last checked on NCBI on December 22nd, 2017). This changed the percentage of outlier sequences from about five to one percent (**Fig. EV1H-K**). We also indicated the sequences that changed in **Appendix Table S1**. Note that the sequences changed on NCBI, but not necessarily on EBI or uniprot.

Appendix Note S2

Single-molecule phenotypes of stalk mutants

Using the single-molecule TIRF assay we observed a variety of phenotypes for all 18 stalk mutations (**Fig. 1B, C**). Two mutants (mutants 15 and 16) did not show any protein expression, suggesting that these mutations may lead to protein misfolding and/or instability (**Fig. EV2A**). Five mutants (mutants 8, 9, 10, 17, and 18) bound microtubules but did not show any single-molecule movement. Many of these mutations are clustered in the proximal region of the stalk, close to the AAA ring (**Fig. EV1L**), and are in regions that are important for interaction with the buttress (Kon *et al*, 2012), suggesting that mutations in these regions severely compromise dynein motility. This is consistent with previous structural work (Schmidt *et al*, 2015; Kon *et al*, 2012) which showed that the interaction between the stalk and buttress plays a key role in dynein motility. However, some 'wild-type' like mutants (mutants 1, 3, 11, and 12) also have insertions or deletions in the vicinity of the stalk and buttress interface but do not seem to impact motility. These mutations are all in CC1 indicating that CC2 might be more sensitive to insertions or deletions in the stalk and buttress interface. One potentially explanation for this observation is that the insertions or deletions in CC2 disturb the previously described kinking of CC2 upon ATP hydrolysis (Schmidt *et al*, 2015) and are therefore more deleterious. In addition, one may expect mutations in the stalk region at the stalk and buttress interface to be accompanied by corresponding mutations in the buttress that have co-evolved to maintain the interaction. However, we did not identify such corresponding mutations.

Overall, our results show that different mutations in overlapping regions on the stalk can result in different phenotypes. For example, the locations of mutations in mutant 4 and mutant 7 overlap with mutant 5 (**Appendix Fig. S1, S2**) very closely, but both these mutants behave more similar to the wild-type protein (**Fig. EV2B**), with clear directional movement and only slightly decreased velocity and processivity (**Fig. EV2B, Appendix Fig. S3**). In the case of mutant 5, mutants 4 and 7 serve as important controls, showing that we have narrowed down an exact region of the stalk that is necessary for allosteric communication. The effect of mutant 5 could be a combination of position, exact length of deletion, and change in structural properties of the new stalk configuration. However, mutants 4 and 7 illustrate that insertions and deletions in overlapping areas can have very small effects on motility.

Similarly, mutants with insertions and deletions containing the same number of residues in the same coiled-coil can move very differently, suggesting that it is not simply the total length of the coil that dictates function. For instance, mutant 1 and mutant 18 have an insertion of 1 residue in CC1, but mutant 1 motility is very similar to wild-type motility, whereas mutant 18 does not move at all. For the three mutants (2, 13, and 15) that have an insertion of 1 residue in CC2, mutant 2 and mutant 13 show diffusive like behavior while mutant 15 was destabilized such that it did not express at all.

Appendix Note S3

Negative stain electron microscopy of mutant 5

Prior to the Cryo-EM study we used a negative stain EM based assay with the goal of assessing the linker conformation and/or flexibility. Due to the large-scale conformational change in the linker, we were previously able to identify distinct linker conformations using 3D classification and refinement from negative stain EM data (Bhabha *et al*, 2014). For this assay, we used the ATP analog AMPPNP to mimic one of the post-force-generating states at AAA1 and AAA3. In this state, the linker in the wild-type enzyme is straight and docked to the AAA ring at AAA5 (Bhabha *et al*, 2014). 3D classification and refinement of negative stain EM data of mutant 5 showed weaker density for the N-terminal region of the linker, suggesting that the position of the linker in mutant 5 may be more flexible than in wild-type (**Appendix Fig. S8A-D**). More strikingly, however, we observed a “gap” (missing density) in the ring at the region of AAA5 (**Appendix Fig. S8B**), in comparison to a more closed state of the wild-type motor (Bhabha *et al*, 2014).

Appendix Tables

**Mutation Number / dimeric or monomeric**	**Strain name**	**Organism**	**NCBI - proteinBLAST**	**EBI - European Bioinformatics Institute**	**Uniprot - HMMER search**
WT / dimer	VY208	Saccharomyces cerevisiae	https://www.ncbi.nlm.nih.gov/protein/767040268?report=genbank&log$=protalign&blast_rank=2&RID=XUTTCCYZ014	-	http://www.uniprot.org/uniprot/P36022
1 / dimer	VY1044	Gorilla gorilla gorilla	UPDATED: https://www.ncbi.nlm.nih.gov/protein/XP_004044002	https://www.ebi.ac.uk/ebisearch/search.ebi?db=allebi&query=ENSGGOP00000020228	http://www.uniprot.org/uniprot/G3RWP4
2 / dimer	VY1045	Helobdella robusta	UPDATED: https://www.ncbi.nlm.nih.gov/protein/675890198?report=genbank&log$=protalign&blast_rank=1&RID=XUU15W9Y014	-	http://www.uniprot.org/uniprot/T1G9C1
3 / dimer	VY1046	Takifugu rubripes	UPDATED: https://www.ncbi.nlm.nih.gov/protein/XP_011616710?report=genbank&log$=protalign&blast_rank=1&RID=41WVKX4Y014	https://www.ebi.ac.uk/ebisearch/search.ebi?db=allebi&query=ENSTRUP00000031696	http://www.uniprot.org/uniprot/H2U434
4 / dimer	VY1047	Branchiostoma floridae	UPDATED (Branchiostoma belcheri): https://www.ncbi.nlm.nih.gov/protein/XP_019639192?report=genbank&log$=protalign&blast_rank=1&RID=41WVXYCX014	-	-
5 / dimer	VY1048	Nasonia vitripennis	UPDATED: https://www.ncbi.nlm.nih.gov/protein/XP_008209982?report=genbank&log$=protalign&blast_rank=1&RID=41WWA4A8014	-	http://www.uniprot.org/uniprot/K7J523

6 / dimer	VY1049	Takifugu rubripes	-	https://www.ebi.ac.uk/ebisearch/search.ebi?db=allebi&query=ENSTRUP00000000144	http://www.uniprot.org/uniprot/H2RJ31
7 / dimer	VY1050	Cavia porcellus	UPDATED: https://www.ncbi.nlm.nih.gov/protein/XP_003463142?report=genbank&log$=protalign&blast_rank=2&RID=41Y4RCPD015	https://www.ebi.ac.uk/ebisearch/search.ebi?db=allebi&query=ENSCPOP00000003676	http://www.uniprot.org/uniprot/H0V2C0
8 / dimer	VY1051	Takifugu rubripes	UPDATED: https://www.ncbi.nlm.nih.gov/protein/XP_003966059?report=genbank&log$=protalign&blast_rank=1&RID=41WXNEUS015	https://www.ebi.ac.uk/ebisearch/search.ebi?db=allebi&query=ENSTRUP00000008414	http://www.uniprot.org/uniprot/H2S7P2
9 / dimer	VY1052	Takifugu rubripes	UPDATED: https://www.ncbi.nlm.nih.gov/protein/XP_003966059?report=genbank&log$=protalign&blast_rank=1&RID=41WY1J2K014	https://www.ebi.ac.uk/ebisearch/search.ebi?db=allebi&query=ENSTRUP00000008415	http://www.uniprot.org/uniprot/H2S7P3
10 / dimer	VY1053	Ciona intestinalis	UPDATED: https://www.ncbi.nlm.nih.gov/protein/XP_018671050?report=genbank&log$=protalign&blast_rank=1&RID=41WYCT79014	https://www.ebi.ac.uk/ebisearch/search.ebi?db=allebi&query=ENSCINP00000008812	-
11 / dimer	VY1054	Ciona savignyi	UPDATED (Ciona intestinalis): https://www.ncbi.nlm.nih.gov/protein/XP_009858173?report=genbank&log$=protalign&blast_rank=1&RID=41WYPZH4015	https://www.ebi.ac.uk/ebisearch/search.ebi?db=allebi&query=ENSCSAVP00000008997	http://www.uniprot.org/uniprot/H2YUI7
12 / dimer	VY1062	Ciona savignyi	UPDATED (Ciona intestinalis): https://www.ncbi.nlm.nih.gov/protein/XP_009858173?report=genbank&log$=protalign&blast_rank=1&RID=41WZ437X015	https://www.ebi.ac.uk/ebisearch/search.ebi?db=allebi&query=ENSCSAVP00000009000	http://www.uniprot.org/uniprot/H2YUJ0
13 / dimer	VY1056	Ciona savignyi	UPDATED (Ciona intestinalis): https://www.ncbi.nlm.nih.gov/protein/XP_018669141?report=genbank&log$=protalign&blast_rank=1&RID=41WZF9S8014	https://www.ebi.ac.uk/ebisearch/search.ebi?db=allebi&query=ENSCSAVP00000010325	http://www.uniprot.org/uniprot/H2YYB4

14 / dimer	VY1057	Ciona intestinalis	-	https://www.ebi.ac.uk/ebisearch/search.ebi?db=allebi&query=ENSCINP00000011393	-
15 / dimer	VY1058	Anolis carolinensis	UPDATED: https://www.ncbi.nlm.nih.gov/protein/XP_003217173?report=genbank&log$=protalign&blast_rank=1&RID=41X056ZX015	https://www.ebi.ac.uk/ebisearch/search.ebi?db=allebi&query=ENSACAP00000016375	http://www.uniprot.org/uniprot/G1KSW2
16 / dimer	VY1059	Gallus gallus	UPDATED: https://www.ncbi.nlm.nih.gov/protein/XP_015137732.1?report=genbank&log$=prottop&blast_rank=8&RID=41X0FWDX015	-	-
17 / dimer	VY1060	E. cuniculi	https://www.ncbi.nlm.nih.gov/protein/19074673?report=genbank&log$=protalign&blast_rank=1&RID=XWEHW0TG015	-	http://www.uniprot.org/uniprot/Q8SR52
18 / dimer	VY1061	Ciona intestinalis	-	https://www.ebi.ac.uk/ebisearch/search.ebi?db=allebi&query=ENSCINP00000011395	-
WT / monomer	VY137	See WT / dimer			
2 / monomer	VY1063	See 2 / dimer			
5 / monomer	VY1065	See 5 / dimer			
13 / monomer	VY1085	See 13 / dimer			

Appendix Table S1. Annotation of all dynein stalk mutant strains used in this study. The VY208 genotype is: MATa; his3-11,15; ura3-1; leu2-3,112; ade2-1; trp1-1; PEP4::HIS5; PRB1D pDyn-pGAL-ZZ-TEV-GFP-3XHA-GST-D6-DYN1-gsDHA:Kan) and the VY137 genotype is: PGal:ZZ:Tev:GFP:HA:D6 MATa; his3-11,15; ura3-1; leu2-3,112; ade2-1; trp1-1; PEP4::HIS5; PRB1D. All sequences that say “UPDATED” do not have any insertions or deletions anymore (based on NCBI (December 22nd, 2017)). For more details see **Appendix Note 1**.

“-” indicates that the sequence was not found (sequence identity less than 60%).

Construct	Nucleotide	K_d [MT]	B_M	k_{basal}
Wild-type	ATP	5.22 ± 0.92 μM	n/m	0.02 ± 0.01
Wild-type	apo	0.78 ± 0.27 μM	0.86 ± 0.02	0.02 ± 0.01
Wild-type	AMPPNP	1.22 ± 0.72 μM	0.90 ± 0.04	0.03 ± 0.01
Mutant 2	ATP	2.62 ± 0.89 μM	n/m	0.01 ± 0.01
Mutant 2	apo	5.83 ± 0.04 μM	n/m	0.01 ± 0.00
Mutant 2	AMPPNP	3.93 ± 1.59 μM	n/m	0.03 ± 0.01
Mutant 5	ATP	4.10 ± 1.28 μM	n/m	0.05 ± 0.00
Mutant 5	apo	5.89 ± 1.38 μM	n/m	0.02 ± 0.01
Mutant 5	AMPPNP	3.08 ± 2.06 μM	n/m	0.02 ± 0.01
Mutant 13	ATP	4.00 ± 0.79 μM	n/m	0.02 ± 0.00
Mutant 13	apo	6.19 ± 0.99 μM	n/m	0.02 ± 0.00
Mutant 13	AMPPNP	3.66 ± 0.08 μM	n/m	0.02 ± 0.00

Appendix Table S2. Microtubule affinity measurements.

The data were fit to the following equation $k_{obs} = (B_M - k_{basal}) \frac{[MT]}{K_d + [MT]} + k_{basal}$ in which B_M is the maximum binding, K_d is the dissociation constant, k_{basal} is the basal "binding" fraction and accounts for the pelleting of dynein without microtubules present, and k_{obs} is the observed fraction of dynein bound (pelleted) over the total amount of dynein. We could have also used the simplified equation $k_{obs} = (B_M) \frac{[MT]}{K_d + [MT]}$ (B_M maximum binding, K_d dissociation constant) but we wanted to account for potential pelleting of dynein without microtubules (k_{basal}). However, since k_{basal} is very low, using the simplified equation gives almost identical results for B_M and K_d. Values are shown as averages of triplicates ± standard deviation. n/m is not measurable.

Mutation	Organism	K_M [MT]	k_{cat}	k_{basal}	Reference
Wild-type	Yeast	0.59 ± 0.28 μM	14.1 ± 0.36 s^{-1}	3.74 ± 0.35 s^{-1}	Cho et al. JCB 2008 (Cho *et al*, 2008)
AAA3 (E2488Q)	Yeast	0.03 ± 0.01 μM	1.38 ± 0.14 s^{-1}	0.30 ± 0.05 s^{-1}	Cho et al. JCB 2008
AAA4 (E2819Q)	Yeast	0.09 ± 0.03 μM	10.6 ± 0.72 s^{-1}	3.36 ± 0.59 s^{-1}	Cho et al. JCB 2008
Wild-type	Yeast	-	20 ± 4 s^{-1}	6 ± 2 s^{-1}	Carter et al. Science 2008 (Carter *et al*, 2011)
Removal of 7 heptads in stalk	Yeast	-	21 ± 2 s^{-1}	13 ± 2 s^{-1}	Carter et al. Science 2008
Insertion of 7 heptads in stalk	Yeast	-	21 ± 5 s^{-1}	6 ± 2 s^{-1}	Carter et al. Science 2008
Wild-type	Yeast	1.06 ± 0.16 μM	16.75 ± 0.49 s^{-1}	3.51 ± 0.31 s^{-1}	Toropova et al. eLife 2014 (Toropova *et al*, 2014)
AAA1	Yeast	-	-	~ 1 s^{-1}	Toropova et al. eLife 2014
AAA5 - linker docking (F3446D, R3445E, K3438E)	Yeast	-	-	~ 2 s^{-1}	Toropova et al. eLife 2014
Wild-type	D.discoideum	33.3 ± 2.6 μM	105.2 ± 4.2 s^{-1}	8.7 ± 0.8 s^{-1}	Kon et al. NSMB 2009 (Kon *et al*, 2009)
Fixed α registry (oxidized)	D.discoideum	5.0 ± 1.0 μM	158.0 ± 1.7 s^{-1}	128.4 ± 6.6 s^{-1}	Kon et al. NSMB 2009
Fixed $\beta+$ registry (oxidized)	D.discoideum	19.2 ± 1.7 μM	17.0 ± 0.6 s^{-1}	3.3 ± 0.1 s^{-1}	Kon et al. NSMB 2009

Fixed β - registry (oxidized)	D.discoideum	20.4 ± 4.6 μM	112.9 ± 4.1 s^{-1}	74.0 ± 2.1 s^{-1}	Kon et al. NSMB 2009
Delta buttress (Q3824-E3864)	D.discoideum	-	-	~ 90 s^{-1}	Kon et al. Nature 2012 (Kon *et al*, 2012)
Delta c-terminus (S4416-I4730)	D.discoideum	-	-	~ 10 s^{-1}	Kon et al. Nature 2012
Wild-type	Yeast	-	~ 17 s^{-1}	~ 3 s^{-1}	Bhabha et al. Cell 2014 (Bhabha *et al*, 2014)
AAA2 - linker docking (A2121G, T2122G, L2123G)	Yeast	-	~ 7 s^{-1}	~ 3 s^{-1}	Bhabha et al. Cell 2014
AAA2 - linker docking (R2183A)	Yeast	-	~ 5 s^{-1}	~ 2 s^{-1}	Bhabha et al. Cell 2014
GST-Dynein (1219-4093)	Yeast	0.39 ± 0.06 μM	16.1 ± 0.3 s^{-1}	-	Reck-Peterson et al. Cell 2006 (Reck-Peterson *et al*, 2006)
Dynein-GST (1219-4093)	Yeast	-	4.3 ± 0.3 s^{-1}	-	Reck-Peterson et al. Cell 2006
GST-Dynein (1390-4093)	Yeast	-	-	~ 1 s^{-1}	Reck-Peterson et al. Cell 2006

Appendix Table S3. ATPase rates for dynein mutations in the literature.

Construct	K_M [MT]	k_{cat}	k_{basal}
Wild-type	0.50 ± 0.17 μM	15.18 ± 1.18 s^{-1}	0.75 ± 0.34 s^{-1}
Mutant 2	n/m	n/m	6.23 ± 2.25 s^{-1}
Mutant 5	n/m	n/m	13.80 ± 0.50 s^{-1}
Mutant 13	n/m	n/m	14.95 ± 0.35 s^{-1}

Appendix Table S4. ATPase assay rate measurements.

The data were fit to the following equation $k_{obs} = (k_{cat} - k_{basal})\frac{[MT]}{K_M+[MT]} + k_{basal}$. Values are shown as averages of triplicates ± standard deviation. n/m is not measurable.

**Data Collection (Cryo-EM)**	**Mutant 5 + AMPPNP**		**Mutant 5 + ATP-vi**	
Microscope	Titan Krios		Arctica	
Camera	K2		K2	
Magnification	22,500		36,000	
Voltage (kV)	300		200	
Electron dose (e-/pixel/second)	10		8	
Focus range (μm)	1.5-3.0		1.5-3.0	
Pixel size (Å)	1.31		1.156	
Number of images/movies	1200		664	
**Reconstruction**				
Particles selected after 2D classification (no.)	310,085		35,565	
CTF correction tool	GCTF 1.0.6		ctffind 4.1.10	
Particle picking method	Gaussian blobs		Gaussian blobs	
Ab-initio models generated (no.)	4		5	
Last round of 3D heterogeneous refinement (no.)	4		5	
Class name	**Class 1**	**Class 2**	**Class 1**	**Class 2 (not shown)**
Point group symmetry	C1	C1	C1	C1
Final particles (no.)	97,008	39,048	8,653	6,629
Resolution (Å)	7.7	7.6	9.2	16.6
B-factor (Å^2)	-400	-400	-400	-400
Modelling from 4W8F domains	rigid-body (Chimera)	rigid-body (Chimera) + refinement (PHENIX)	rigid-body (Chimera)	None

Appendix Table S5. Statistics on cryo-EM data collection and processing.

References

Alva V, Nam S-Z, Söding J & Lupas AN (2016) The MPI bioinformatics Toolkit as an integrative platform for advanced protein sequence and structure analysis. *Nucleic Acids Res.* **44:** W410–5

Bhabha G, Cheng H-C, Zhang N, Moeller A, Liao M, Speir JA, Cheng Y & Vale RD (2014) Allosteric communication in the dynein motor domain. *Cell* **159:** 857–868

Carter AP, Cho C, Jin L & Vale RD (2011) Crystal structure of the dynein motor domain. *Science* **331:** 1159–1165

Carter AP, Garbarino JE, Wilson-Kubalek EM, Shipley WE, Cho C, Milligan RA, Vale RD & Gibbons IR (2008) Structure and functional role of dynein's microtubule-binding domain. *Science* **322:** 1691–1695

Cho C, Reck-Peterson SL & Vale RD (2008) Regulatory ATPase sites of cytoplasmic dynein affect processivity and force generation. *J. Biol. Chem.* **283:** 25839–25845

Edelstein A, Amodaj N, Hoover K, Vale R & Stuurman N (2010) Computer control of microscopes using µManager. *Curr. Protoc. Mol. Biol.* **Chapter 14:** Unit14.20

Katoh K, Misawa K, Kuma K-I & Miyata T (2002) MAFFT: a novel method for rapid multiple sequence alignment based on fast Fourier transform. *Nucleic Acids Res.* **30:** 3059–3066

Kon T, Imamula K, Roberts AJ, Ohkura R, Knight PJ, Gibbons IR, Burgess SA & Sutoh K (2009) Helix sliding in the stalk coiled coil of dynein couples ATPase and microtubule binding. *Nat. Struct. Mol. Biol.* **16:** 325–333

Kon T, Oyama T, Shimo-Kon R, Imamula K, Shima T, Sutoh K & Kurisu G (2012) The 2.8 [thinsp] A crystal structure of the dynein motor domain. *Nature* **484:** 345–350

Pettersen EF, Goddard TD, Huang CC, Couch GS, Greenblatt DM, Meng EC & Ferrin TE (2004) UCSF Chimera—A visualization system for exploratory research and analysis. *J. Comput. Chem.* **25:** 1605–1612

Reck-Peterson SL, Yildiz A, Carter AP, Gennerich A, Zhang N & Vale RD (2006) Single-molecule analysis of dynein processivity and stepping behavior. *Cell* **126:** 335–348

Schmidt H, Zalyte R, Urnavicius L & Carter AP (2015) Structure of human cytoplasmic dynein-2 primed for its power stroke. *Nature* **518:** 435–438

Tomishige M, Stuurman N & Vale RD (2006) Single-molecule observations of neck linker conformational changes in the kinesin motor protein. *Nat. Struct. Mol. Biol.* **13:** 887–894

Toropova K, Zou S, Roberts AJ, Redwine WB, Goodman BS, Reck-Peterson SL & Leschziner AE (2014) Lis1 regulates dynein by sterically blocking its mechanochemical cycle. *Elife* **3:** Available at: http://dx.doi.org/10.7554/eLife.03372

Zheng SQ, Palovcak E, Armache J-P, Verba KA, Cheng Y, Agard DA. 2017. MotionCor2:

anisotropic correction of beam-induced motion for improved cryo-electron microscopy. *Nat Methods* **14**:331–332.
