# Supplementary material for: Coupling of ATPase activity, microtubule binding, and mechanics in the dynein motor domain
Source: EMBO J. 2019 May 31;38(13):e101414. doi: 10.15252/embj.2018101414 (PMC6600642; doi:10.15252/embj.2018101414)
Supplement: Supplementary file 2 — Expanded View Figures PDF [file EMBJ-38-e101414-s002.pdf]

Expanded View Figures

Figure EV1. Sequence analysis and conservation in the dynein motor domain.

A Conserved residues in the stalk–buttress interface of dynein mapped onto the structure of the human cytoplasmic dynein 2 [PDB: 4RH7 (Schmidt *et al*, 2015)]. Note, the amino acids listed are based on numbering from yeast cytoplasmic dynein 1.

B, C The length (number of residues) conservation (B) and the sequence conservation (C) of domains of the dynein motor domain are shown. The conservation score is derived from Jalview (Livingstone & Barton, 1993; Waterhouse *et al*, 2009) and is shown for each domain. The conservations are based on 534 different sequences that were curated as described in Appendix Note S1.

D–G Sequence conservation (D), conservation of hydrophobic residues (E), charge variation (F) (how many residues at the same position among different sequences switch between D/E and H/K/R), and conservation of charge (G) where basic residues (D/E) are in blue and acidic residues (H/K/R) are in red. The conservations shown are based on 534 different sequences that were curated as described in Appendix Note S1.

H, I Histogram showing the length distribution of CC1 (H) and CC2 (I) of the dynein stalk among 534 sequences with initial sequence data (used to derive mutants) as described in Appendix Note S1. Orange box indicates area that is magnified on the right.

J, K Histogram showing the length distribution of CC1 (J) and CC2 (K) of the dynein stalk among 534 sequences that were updated based on most recent sequencing reads in various databases as described in Appendix Note S1. Orange box indicates area that is magnified on the right, showing a handful of outlier sequences with different stalk lengths.

L Coverage of all mutations (red) generated in the yeast dynein background based on our sequence analysis (most left) mapped onto the structure of human cytoplasmic dynein 2 stalk [PDB: 4RH7(Schmidt *et al*, 2015)]. Regions of individual mutants are shown in Appendix Fig S2. Positions of insertions/deletions that showed "Diffusive-like" (orange) movement, "No movement/No expression" (black), "Directional" (green), and "transient binding" (blue) mapped onto the stalk (classification as shown in Fig 1).

 © 2019 The Authors

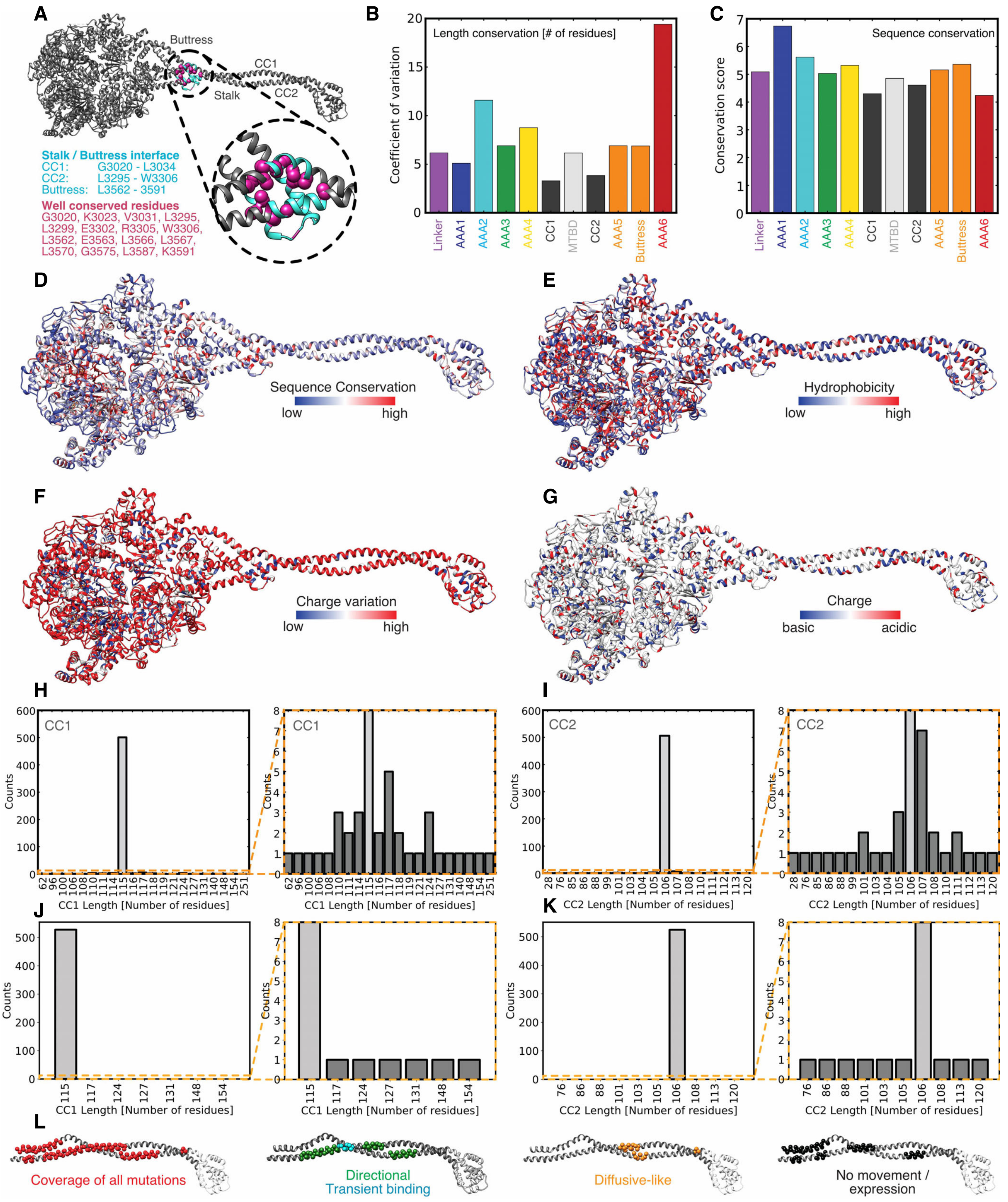
A
Buttress
CC1
Stalk
CC2
Stalk / Buttress interface
CC1: G3020 - L3034
CC2: L3295 - W3306
Buttress: L3562 - 3591
Well conserved residues
G3020, K3023, V3031, L3295, L3299, E3302, R3305, W3306, L3562, E3563, L3566, L3567, L3570, G3575, L3587, K3591
B
Length conservation [# of residues]
Coefficient of variation
Linker AAA1 AAA2 AAA3 AAA4 CC1 MTBD CC2 AAA5 Buttress AAA6
C
Sequence conservation
Conservation score
D
Sequence Conservation
low high
E
Hydrophobicity
low high
F
Charge variation
low high
G
Charge
basic acidic
H
CC1
Counts
CC1 Length [Number of residues]
I
CC2
CC2 Length [Number of residues]
J
K
L
Coverage of all mutations
Directional
Transient binding
Diffusive-like
No movement / expression

Figure EV1.

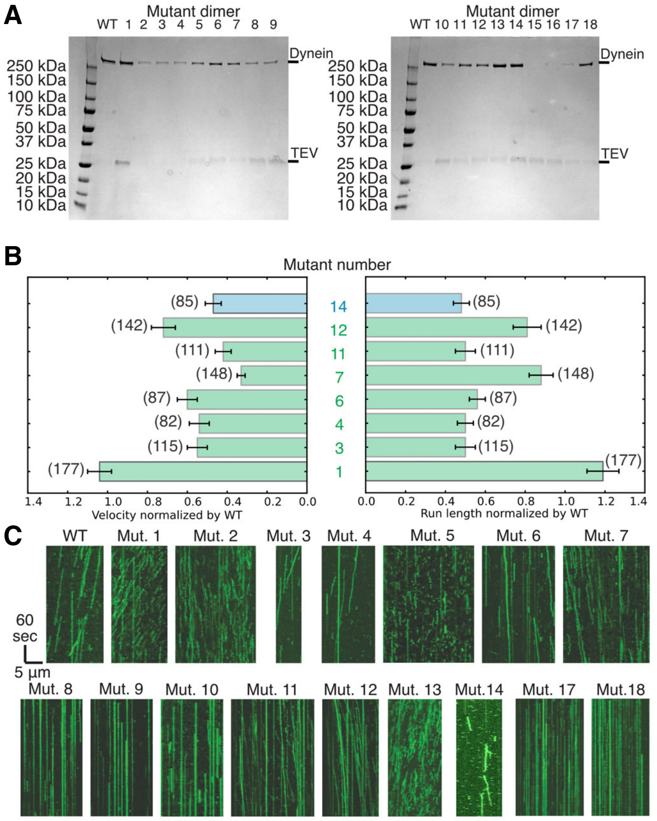

Figure EV2. Example kymographs and single-molecule motility properties of stalk mutants.

A Purified dynein, wild-type, and mutants, after affinity purification shown by PAGE. No dynein band is visible for mutants 15 and 16 indicating that they did not express. For some constructs, residual TEV, which was used the cleave the dynein of beads during the affinity purification (see Materials and Methods), is visible. All constructs that were used for assays other than the single-molecule motility assay were further purified by size exclusion chromatography, which removed the residual TEV entirely (see Materials and Methods).

B Velocity and run length of "Directional" motors (green—mutants 1, 3, 4, 6, 7, 11, and 12) and the "transient binding" motor (blue—mutant 14) normalized by wild-type dynein. Error bars show standard deviation, and number in brackets indicates the number of motors quantified. Data used for quantification are shown in Appendix Fig S3.

C Example kymographs showing different types of movement as classified in Fig 1. Kymographs for mutants 15 and 16 are not shown since they did not express.

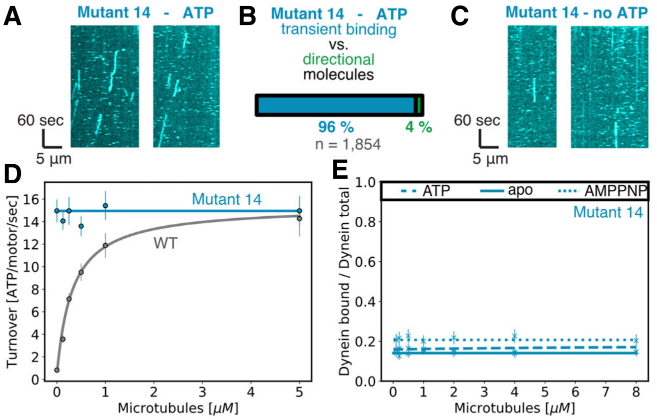

Figure EV3. Biochemical properties of mutant 14.

A Kymographs of mutant 14 from single-molecule assay with 1 mM ATP.

B Quantification of percentage of transient binding or directionally moving motors of mutant 14 in the presence of ATP.

C Kymographs of mutant 14 from single-molecule assay without ATP.

D Microtubule-stimulated ATPase activity of mutant 14 (cyan) and wild-type (gray) dynein. Error bars show standard deviation of three repetitions of different dynein preparations. Caption for Appendix Table S4 shows fit equation for ATPase data. The basal ATPase rate k_{basal} for mutant 14 is 14.51 ± 0.27/s while k_{cat} and [MT] K_M were not measurable. For wild-type dynein, we measured a k_{cat} of 15.41 ± 1.31/s, [MT] K_M of 0.42 ± 0.10 µM, and k_{basal} of 0.42 ± 0.33/s which is in good agreement with the measurements shown in Fig 2E and F, and Appendix Table S4.

E Microtubule affinity measured by a cosedimentation assay in the apo state (full line) and in the presence of ATP (dashed line), and AMPPNP (dotted line) for mutant 14. Error bars show standard deviation of three repetitions of different dynein preparations. Caption for Appendix Table S2 shows fit equation for microtubule affinity data. The maximum binding (B_M) and the dissociation constant K_d were not measurable for mutant 14 in all three nucleotide states.

 © 2019 The Authors

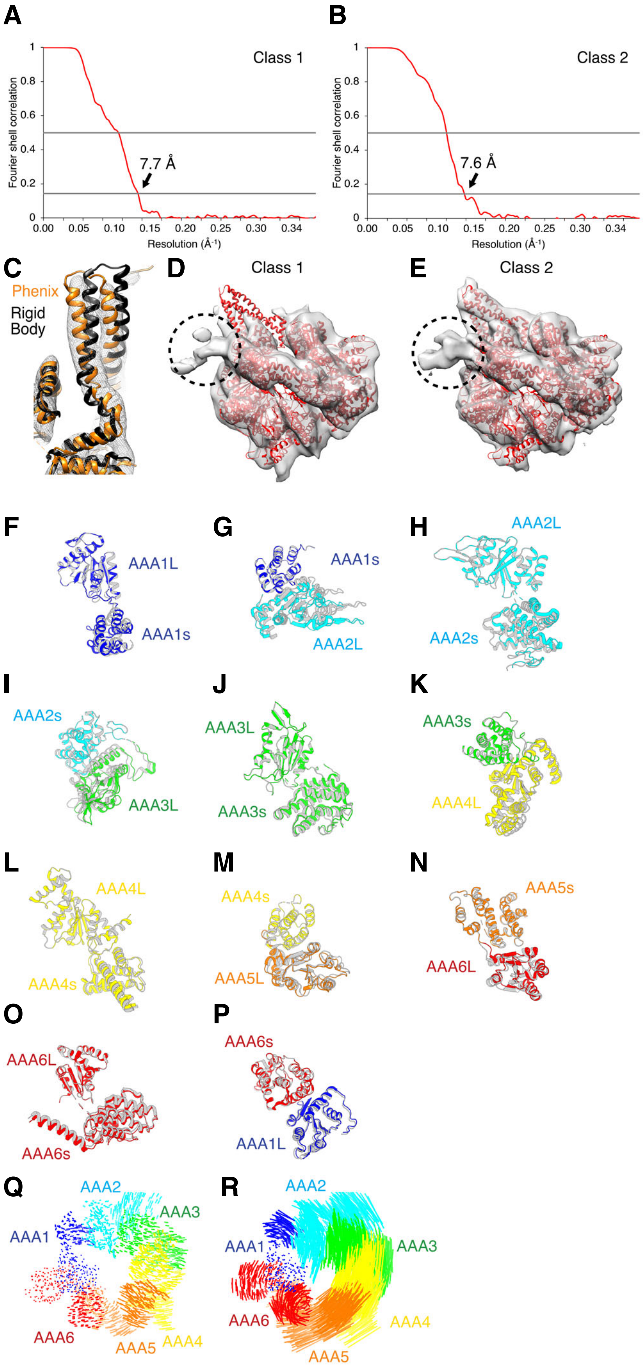

Figure EV4. Cryo-EM analysis for class 1 and class 2 of mutant 5 in the presence of AMPPNP.

A, B Plot of Fourier shell correlation (FSC) for class 1 (A) and class 2 (B). Local resolution estimation of the mutant 5 density maps in AMPPNP is shown in Appendix Fig S7.

C Cryo-EM density for the buttress region of class 2 with rigid body and flexibly fit models. The rigid body fit (black) of AAA5L into the density clearly showed that some rearrangement of the buttress had occurred. Flexible fitting in Phenix (orange) resulted in a model that fit the density in the buttress region significantly better.

D, E Cryo-EM reconstruction of class 1 (D) and class 2 (E) showing unfiltered maps with AMPPNP-bound crystal structure (PDB: 4W8F, red) shown for reference. Black-dotted circle indicates position of a GFP tag at the N-terminus of the linker, which is better defined in class 2.

F–P Domain movements between class 1 and class 2 of mutant 5 in AMPPNP cryo-EM data. In every panel, the top domain is aligned, showing movement between that domain and the next. Class 1 is colored, and class 2 is gray. Movement between AAA1L and AAA1s (F). Movement between AAA1s and AAA2L (G). Movement between AAA2L and AAA2s (H). Movement between AAA2s and AAA3L (I). Movement between AAA3L and AAA3s (J). Movement between AAA3s and AAA4L (K). Movement between AAA4L and AAA4s (L). Movement between AAA4s and AAA5L (M). Movement between AAA5s and AAA6L (N). Movement between AAA6L and AAA6s (O). Movement between AAA6s and AAA1L (P).

Q Visualization of interalpha carbon distances between class 1 and class 2 of mutant 5 in the AMPPNP state after alignment on AAA1L as seen from the top. We removed the linker for clarity.

R Visualization of interalpha carbon distances between class 1 of mutant 5 in the AMPPNP state and the cryo-EM model of yeast dynein in the presence of ADP-vi (Bhabha *et al*, 2014). We removed the linker for clarity.

Figure EV5. Cryo-EM analysis of mutant 5 in the presence of ADP-vanadate.

A Plot of Fourier shell correlation (FSC) for mutant 5 in the presence of ADP-vanadate.

B Cryo-EM reconstruction of mutant 5 with ADP-vanadate (gray) fitted with models of human cytoplasmic dynein 2 in the ADP-vi state [left—PDB: 4RH7 (Schmidt *et al*, 2015)], yeast cytoplasmic dynein mutant 5 in ADP-vi state (middle—this study), and yeast cytoplasmic dynein in the AMPPNP state [right—PDB: 4W8F (Bhabha *et al*, 2014)]. For the mutant 5 ADP-vi state, only the part of the linker with sufficient density was fitted.

C Closure of AAA1L and AAA2L domains in wild-type dynein. Same view of the AAA1 and AAA2 interface as shown in Fig 4B. Left: Structures of the human cytoplasmic dynein 2 in the ADP-vi state [green—PDB: 4RH7 (Schmidt *et al*, 2015)] and the yeast cytoplasmic dynein in the AMPPNP state [blue—PDB: 4W8F (Bhabha *et al*, 2014)] with axes that were fit through the following residues: The gray AAA1L axis is defined with residues taken from the yeast structure using Walker-A (K1802), Walker-B (D1848), and Q1829. The green wild-type ADP-vi AAA2L axis is defined by the arginine finger (R2109), the Sensor-II (R1867), and a residue from the insert loop (G2020). The blue wild-type AMPPNP AAA2L axis is defined by the arginine finger (R2209), the Sensor-II (R1971), and a residue from the insert loop (G2116). Right: Merge between the two representations on the left. The angle between the green and blue axes was calculated using Chimera (Pettersen *et al*, 2004). The structures were aligned on AAA1L.

D Closure of AAA1L and AAA2L domains in mutant 5 dynein. Same view of the AAA1 and AAA2 interface as shown in Fig 4B. Left: Structures of the yeast cytoplasmic dynein mutant 5 in ADP-vi state (orange—this study) and the yeast cytoplasmic dynein mutant 5 in AMPPNP—class 1 state (purple—this study) with axes that were fit through the following residues: The gray AAA1L axis is defined with residues taken from the yeast structure using Walker-A (K1802), Walker-B (D1848), and Q1829. The orange mutant 5 ADP-vi AAA2L axis is defined by the arginine finger (R2209), the Sensor-II (R1971), and a residue from the insert loop (G2116). The purple mutant 5 AMPPNP AAA2L axis is defined by the arginine finger (R2209), the Sensor-II (R1971), and a residue from the insert loop (G2116). Right: Merge between the two representations on the left. The angle between the orange and purple axes was calculated using Chimera (Pettersen *et al*, 2004). The structures were aligned on AAA1L.

E Approximate distances between key regions of the ATP-binding sites in wild-type and mutant 5 dynein structures. Close-up view of ADP-vi-binding pocket in AAA1. The structures of (from left to right) yeast cytoplasmic dynein in the AMPPNP state [blue—PDB: 4W8F (Bhabha *et al*, 2014)], human cytoplasmic dynein 2 in the ADP-vi state [green—PDB: 4RH7 (Schmidt *et al*, 2015)], yeast cytoplasmic dynein mutant 5 in AMPPNP—class 1 state (purple—this study), and yeast cytoplasmic dynein mutant 5 in ADP-vi state (orange—this study) were aligned on AAA1L. ADP and vanadate are depicted in black or gray and taken from the human cytoplasmic dynein 2 structure. The positions of the arginine finger (RF—human structure: R2109; yeast structures: R2209), the Walker-A (WA—human structure: K1695; yeast structures: K1802), the Walker-B (WB—human structure: D1741; yeast structures: D1848), and the Sensor-II (S-II—human structure: R1867; yeast structures: R1971) are shown in non-opaque colors for the respective structures. For each structure, we calculated the approximate distance between the alpha carbon of the arginine finger (RF) and the Walker-A (WA) motif using Chimera (Pettersen *et al*, 2004) as an approximation for the closure of the nucleotide-binding pocket.

F ATPase activity of wild-type and mutant 5 at different concentrations of vanadate (0–100 μM). The turnover rate of both wild-type (gray) and mutant 5 (orange), is reduced as the concentration of vanadate increases indicating that vanadate binds to the AAA1 nucleotide-binding pocket of wild-type as well as mutant 5. The x and the dot are measurements of two technical repeats. The solid line connects the average values of the two technical repeats. Dashed box shows the area that is shown in (G). All measurements were performed in the presence of 1 mM Mg-ATP and 6 μM microtubules (MTs).

G Same as in (F) but for vanadate concentrations from 0 to 10 μM.

H Vanadate-mediated UV photo-cleavage of mutant 5 in the presence of 2 mM Mg-ATP and 2 mM vanadate. The arrows show two bands of ~ 270 and ~ 90 kDa after exposure to ultraviolet light (+UV) which suggests that vanadate binds to the AAA1 nucleotide-binding pocket of mutant 5.

I Domain movements between mutant 5 and wild-type [PDB: 4RH7 (Schmidt *et al*, 2015)] in the presence of ADP-vi. The two structures were aligned on AAA1L [matchMaker in Chimera (Pettersen *et al*, 2004)].

J Domain movements between mutant 5 in the presence of ADP-vi and—from left to right—wild-type in the ADP-vi state [PDB: 4RH7 (Schmidt *et al*, 2015)], wild-type in the AMPPNP state [PDB: 4W8F (Bhabha *et al*, 2014)], class 1 of mutant 5 with AMPPNP, and wild-type in the apo state [PDB: 4AKG (Schmidt *et al*, 2012)]. For every structure, the domains are aligned on AAA5L [matchMaker in Chimera (Pettersen *et al*, 2004)].

K Linker of wild-type motor in the presence of AMPPNP [PDB: 4W8F (Bhabha *et al*, 2014)] is shown in purple with AAA5L domain in the ADP-vi state of wild-type [gray—PDB: 4RH7 (Schmidt *et al*, 2015)] and mutant 5 (orange). All structures were aligned on AAA2L. While there is a visible clash between the AMPPNP linker and the AAA5L domain of wild-type in the presence of ADP-vi, there is no clash of the linker with the AAA5L of mutant 5 in ADP-vi. This observation might explain why the linker in wild-type dynein bends in the presence of ADP-vanadate and why it remains straight for mutant 5.

© 2019 The Authors

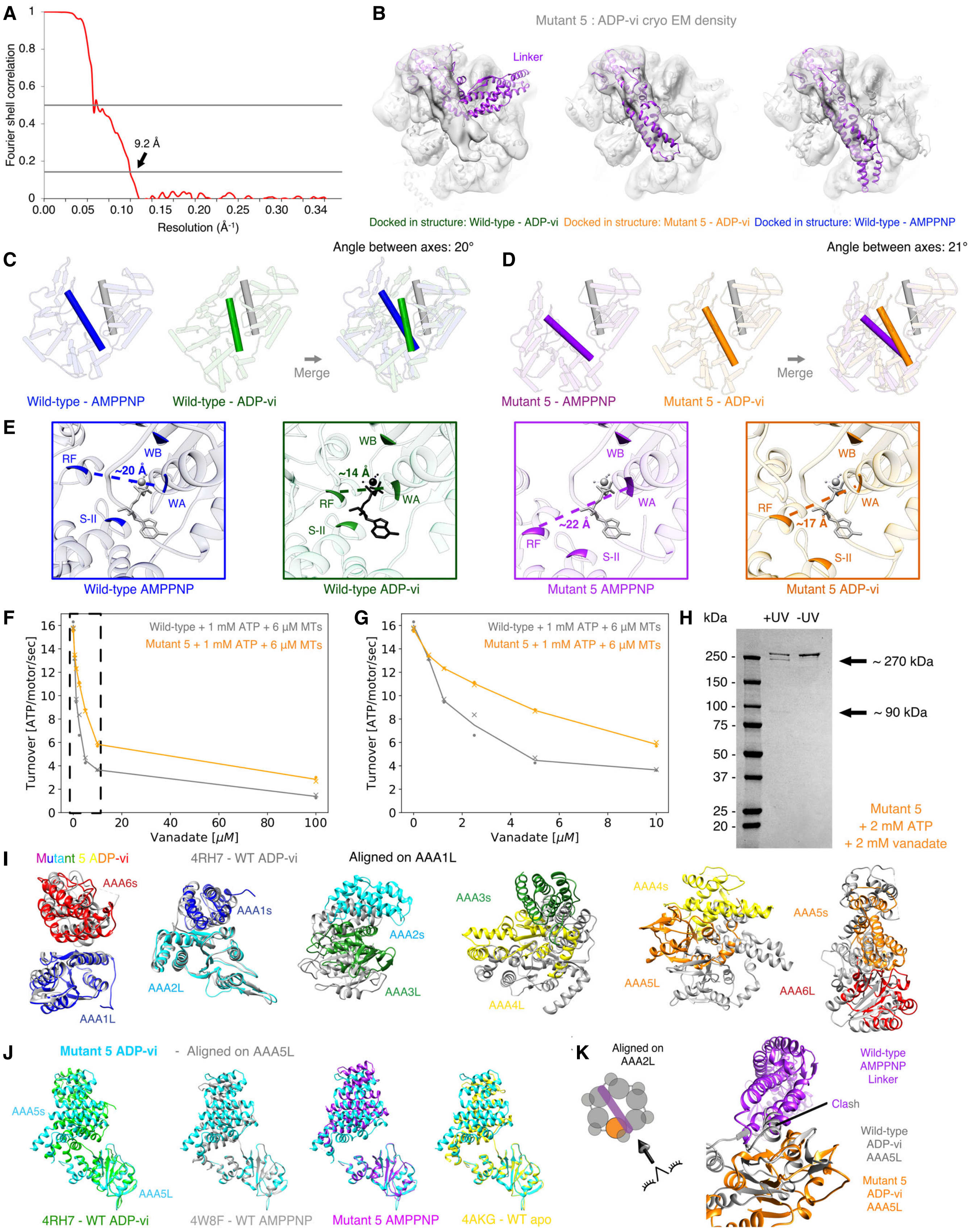

Figure EV5.

© 2019 The Authors
